# Onlays/partial crowns versus full crowns in restoring posterior teeth: a systematic review and meta-analysis

**DOI:** 10.1186/s13005-022-00337-y

**Published:** 2022-11-21

**Authors:** Bingjie Wang, Jiayan Fan, Lutao Wang, Bin Xu, Liang Wang, Luyi Chai

**Affiliations:** 1 Department of Stomatology, The Affiliated People’s Hospital of Ningbo University (Ningbo Yinzhou People’s Hospital), No.251, Baizhang Road(E), Ningbo, 315000 China; 2 Department of Stomatology, Ningbo No.2 Hospital, Ningbo, 315000 China

**Keywords:** Onlays, Partial crowns, Crowns, Survival, Success

## Abstract

**Background:**

Tooth-colored onlays and partial crowns for posterior teeth have been used increasingly in clinics. However, whether onlays/partial crowns could perform as well as full crowns in the posterior region was still not evaluated thoroughly.

**Methods:**

A literature search was conducted without language restrictions in Pubmed, Embase, Cochrane Central Register of Controlled Trial and Web of science until September 2021. RCTs, prospective and retrospective observational studies with a mean follow-up of 1 year were selected. Cochrane Collaboration’s tool was adopted for quality assessment of the RCT. The quality of observational studies was evaluated following Newcastle-Ottawa scale. The random-effects and fixed-effects model were employed for meta-analysis.

**Results:**

Four thousand two hundred fifty-seven articles were initially searched. Finally, one RCT was identified for quality assessment and five observational studies for qualitative synthesis and meta-analysis. The RCT was of unclear risk of bias while five observational studies were evaluated as low risk. The meta-analysis indicated no statistically significant difference in the survival between onlays/partial crowns and full crowns after 1 year (OR = 0.55, 95% CI: 0.02-18.08; I^2^ = 57.0%; *P* = 0.127) and 3 years (OR = 0.65, 95% CI: 0.20-2.17; I^2^ = 0.0%; *P* = 0.747). For the success, onlays/partial crowns performed as well as crowns (OR = 0.58, 95% CI: 0.20-1.72; I^2^ = 0.0%; *P* = 0.881) at 3 years. No significant difference of crown fracture existed between the two methods (RD = 0.00, 95% CI: − 0.03-0.03; I^2^ = 0.0%; *P* = 0.972).

**Conclusions:**

Tooth-colored onlays/partial crowns performed as excellently as full crowns in posterior region in a short-term period. The conclusions should be further consolidated by RCTs with long-term follow-up.

**Supplementary Information:**

The online version contains supplementary material available at 10.1186/s13005-022-00337-y.

## Background

Recently, with the rapid development of dental adhesive technology, tooth-colored onlays and partial crowns for posterior teeth have been increasingly used in contemporary restorative dentistry [1]. Onlay, partial coverage restoration of a tooth retained by conventional and resin cements that restores one or more cusps as well as the partial or entire occlusal surface [2], is increasingly accepted by dentists due to its advantages. In the cases of a large amount of tooth structure loss, onlays possess advantages of better re-establishment of tooth contours, resulting in more proper function as well as protection the weakened tooth at the same time when compared with direct filling restorations and inlays [3]. Full crowns usually create satisfactory occlusal and proximal anatomy [4]. However, conventional crowns sacrifice amounts of residual sound hard tissues [5]. Edelhoff & Sorensen [5] reported that the preparations of full crowns were the most invasive with 67.5 to 75.6% tooth structure removal while onlays/partial crowns only removed 35.5 to 46.7% tooth structure.

Clinical trials have confirmed the promising performance of onlays/partial crowns in the posterior region [6–10]. Federlin et al. [6] investigated the clinical efficiency of partial ceramic crowns (PCCs) and revealed the cumulative survival rate of 88.8% for PCCs after 5.5-year observation in stress-bearing posterior teeth. Özyoney et al. [7] reported that IPS Empress II (Ivoclar Vivadent, Schaan, Liechtenstein) onlays demonstrated promising results with the success rate of 92.5% after 4-year observation period. Archibald et al. [8] revealed that the estimated survival rate of IPS e.max (Ivoclar Vivadent, Schaan, Liechtenstein) onlays reached 96.3% after 2 years and 91.5% at 4 years. Vagropoulou et al. [9] systematically reviewed the clinical trials published between 1980 and 2017, showing that the cumulative survival rate of onlays was 93.50% after 5 years of follow-up. In the meanwhile, Abduo & Sambrook [10] also evaluated the clinical outcomes of ceramic onlays systematically and demonstrated that the medium- (2-5 years) and long-term (more than 5 years) survival rates of ceramic onlays were 91-100% and 71-98.5%, indicating that ceramic onlays appeared to be alternatives to classical full crowns in restoring posterior teeth.

As a traditional and reliable method restoring the posterior teeth, full crowns are used extensively in clinics worldwide when teeth are destroyed severely [11–13], especially when the loss of tooth structure exceeds 50% [14]. Actually, a number of studies have proved that full crowns have been achieved reliable results in restoring the defected teeth regardless of the restorative materials [15–19]. Kassardjian et al. [17] revealed that 90.9% of all-ceramic crowns in posterior region had satisfactory performance with a follow-up time of 36-223 months. Aziz et al. [19] evaluated the longevity of chairside monolithic lithium disilicate crowns and demonstrated that the cumulative rates of the survival and success were 95 and 92.3% after 4 years. Larsson & wennerberg [16] reported that according to life table and analysis, the 5-year cumulative survival rate of the zirconia crowns reached 95.9%. In addition, Pjetursson et al. [15] also indicated that the estimated survival rates of metal-ceramic crowns and all-ceramic crowns were 95.6 and 93.3% at 5-year recall appointment, respectively. Van den breemer et al. [18] revealed that the cumulative survival rates of monolithic lithium disilicate crown restorations were 92, 85.5 and 81.9% after 5, 10 and 15 years.

Numerous laboratory investigations and clinical trials have demonstrated that the preservation of sound tooth structure is a significant ingredient for the durability of the restorations and teeth [20–25]. Based on the studies and concept, onlays/partial crowns will play an increasingly important role in restorative dentistry [12]. Several laboratory studies and clinical trials have compared the performance of onlays/partial crowns with full crowns in stress-bearing posterior teeth [26–29]. However, the comparison between the two types of restorations has not been thoroughly evaluated. It is difficult and confused to decide which type of restoration should be employed to restore the defected teeth in posterior region at times.

With the increasing usage of onlays/partial crowns, the aim of this review and meta-analysis was to systematically compare the survival and success of onlays/partial crowns with classical full crowns as well as analyze the complications of both groups after 1, 3 and 6.6 years follow-up.

## Methods

The meta-analysis was carried out following the PRISMA statement [30]. The systematic review protocol was registered under number CRD42022277014 on the PROSPERO database.

### Eligibility criteria

The search strategy performed for the systematic review was on the basis of the elements of PICOS:

- P (Population): adult patients with onlays/partial crowns or full crowns in the posterior teeth.
- I (Intervention): adult patients with onlays/partial crowns in the posterior teeth.
- C (Comparison): adult patients with full crowns in the posterior teeth.
- (Outcome): Primary outcome included Odds ratios for onlays/partial crowns and full crowns regarding the survival rate and success rate; Secondary outcome contained risk difference of predominant complications for the two different restorative methods.
- S (Study): RCTs, prospective or retrospective observational study with at least 1-year of follow-up were identified in the review.

### Exclusion criteria

1. Reviews, case series, case reports, letters, laboratory studies, animal studies, and meeting abstracts.
2. If the same population was involved in two or more articles, the most recent study was selected.

### Search strategy

PubMed, Embase, Cochrane Central Register of Controlled Trial and Web of science were searched until September 13, 2021. Additionally, the website ClinicalTrials.gov (www.clinicaltrial.gov) was also searched to identify ongoing or unpublished clinical trials related with the topic of review.

The following search strategy was conducted on PubMed as an example:

- #1. onlay OR partial coverage restoration OR partial crown OR occlusal veneer
- #2. crown OR full coverage restoration OR complete coverage restoration
- #3. clinical performance OR clinical study OR follow-up study OR retrospective study OR prospective study OR survival rate OR success rate OR clinical outcome
- #4. #1 AND #2 AND #3

Endnote X9 software was operated to eliminate duplicates after all of the relevant articles were imported. Two reviewers screened and evaluated all titles and abstracts, independently. Full texts of the abstracts with insufficient information and potential articles were reading and assessed carefully according to the eligibility criteria. Any disagreement was resolved by discussion by the two reviewers.

### Data extraction

The data was extracted from the selected studies by two reviewers independently. The following information was obtained from each identified trial by the reviewers: authors (year), materials/methods, cement, country, Investigation period, evaluation criteria, follow-up period, age range (mean), study type, number of patients, dropout of restorations (%), number of onlays/full crowns, failures of onlays/full crowns, defects of onlays/full crowns, score of Newcastle-Ottawa Scale (NOS). Any doubts and discrepancies on data extracting were resolved by data rechecking and discussion. When the two reviewers did not agree with each other, discrepancies would be settled and arbitrated by a third reviewer.

### Quality assessment

The quality of the identified RCT was assessed by two reviewers independently and in duplicate in accordance with the Cochrane Risk of Bias tool [31]. The tool evaluated random sequence generation, allocation concealment, blinding of participants and personnel, blinding of outcome assessment, incomplete outcome data, selective reporting and other bias [31]. The quality analysis of the identified observational studies was carried out following the Newcastle-Ottawa Scale (NOS) [32]. The studies were dichotomized into high quality and low quality according to the aspects of the quality of selection, comparability and outcome. A study scoring no less than 6 was considered to be of high methodological quality, while a study scoring less than 6 was considered to be low quality. Any disagreement and doubts between two reviewers were resolved by discussion.

### Measures and statistical analyses

Interrater reliability was assessed by using Cohen’s Kappa coefficient test at the title/abstract screening and full-text review stages. Descriptive and statistical analyses were conducted to compare the clinical efficiency of onlays/partial crowns with full crowns. Odds ratio (OR) with a confidence interval (CI) of 95% was adopted for the effects of intervention. In addition, we also computed the risk difference (RD) for the main complications of both groups. Inconsistency index (I^2^) statistic and Q statistic were employed for analyzing heterogeneity. The data was analyzed using the fixed-effects model if no heterogeneity of the eligible studies existed. Otherwise, the random-effects model was employed. Visually assessing the symmetry of Begg’s funnel plots and Egger’s test were adopted for the possibility of publication bias. *P* value less than 0.05 was considered statistically significant. STATA, version 15.0 (Stata corp., College station, TX, USA) was operated for all statistical analyses.

## Results

### Literature search

**Fig. 1:**
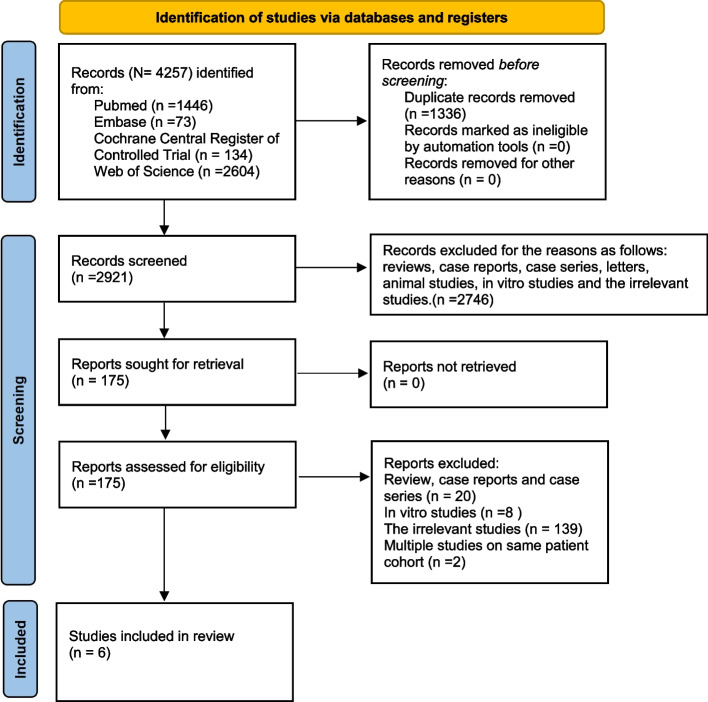
The flowchart of the search strategy.

A total of 4257 articles was identified after initial searches in the databases mentioned above and no additional study ongoing or unpublished was obtained. 1336 studies were removed due to duplicates. 2746 articles were eliminated after all the titles and abstracts were screened while 175 were remained for full-text assessment. At last, 169 studies were ruled out. Six studies [27, 33–37] were included for qualitative synthesis and five studies [27, 33–35, 37] for meta-analysis in this review. The screening process of study identification is presented in Fig. 1. The K agreement between the two reviewers was 0.85 at the screening and 0.90 at the full-text review stage.

### Characteristics of the included studies

The main information and characteristics of the identified studies are listed in Table 1. Five studies [27, 33–35, 37] included in this review were clinical observational trials with at least 3 years of follow-up. Only one study [36] was RCT with 1 year of follow-up. Four studies [33, 35–37] compared the clinical performance of ceramic onlays/partial crowns with full crowns fabricated from lithium disilicate (IPS e.max) or Finess all-ceramic (Dentsply Ceramco, Burlington, NJ, USA). One study [34] investigated the efficacy of the composite resin onlays in comparison with crowns. Another study [27] involved two different materials, investigating the effectiveness of IPS e.max onlays and porcelain-fused-metal (PFM) crowns. Among the selected studies, the RCT [36] was adopted for quality assessment but not for meta-analysis due to the different study type from other studies.

**Table 1:** Characteristics of the included studies

No.	Author (year)	Materials / Methods	Cement	Country	Investigation Period	Evaluation criteria	Follow-up period (y)	Age range (mean)	Study type	No of Patients	Dropout of restorations (%)	No of onlays / crowns	Failures of onlays / crowns	Defects of onlays / crowns	score
1	Li et al. (2015) [27]	IPS Emax press / pressable PFM (gold) / casting	Adhesive	China	2010.2-2010.11	customized criteria	3	21-88 (42)	PC	94	0	46 / 80	1 / 2	2 / 5	8
2	Barnes et al. (2010) [33]	Finess all-ceramic / pressable	Adhesive	NR	NR	Modified Rage criteria	3	NR	prospective study	NR	31.60%	19 / 9	1 / 0	1 / 0	6
3	Jongsma et al. (2012) [34]	Neco / photopolymerized	Adhesive / Conventional	Nethelans	NR	customized criteria	3	29-70 (53)	PC	45	0	5 / 86	0 / 7	0 / 15	7
4	Fabbri et al. (2014) [35]	IPS Emax press / pressable	Adhesive	Italy	2006.6-2010.12	Modified CDA criteria	3	19-71	retrospective study	NR	0	62 / 197	1 / 7	1 / 8	7
5	Li et al. (2019) [36]	IPS Emax cad / CAD/CAM	Adhesive	China	2016.5-2016.12	Modified USPHS	1	67.5	RCT	66	5.30%	38 / 40	0 / 0	3 / 5	/
6	Fotiadou et al. (2021) [37]	IPS Emax press / pressable	Adhesive	German	2010.1-2014.1	FDI criteria	6.6	26-84 (59)	RC	145	0	179 / 5	7 / 1	NR	7

*USPHS* United States Public Health Service, *CDA* California Dental Association, *NR* not reported, *PC* prospective cohort, *RC* retrospective cohort, Score the value of Newcastle-Ottawa Scale (NOS)

### Quality assessment

**Fig. 2:**
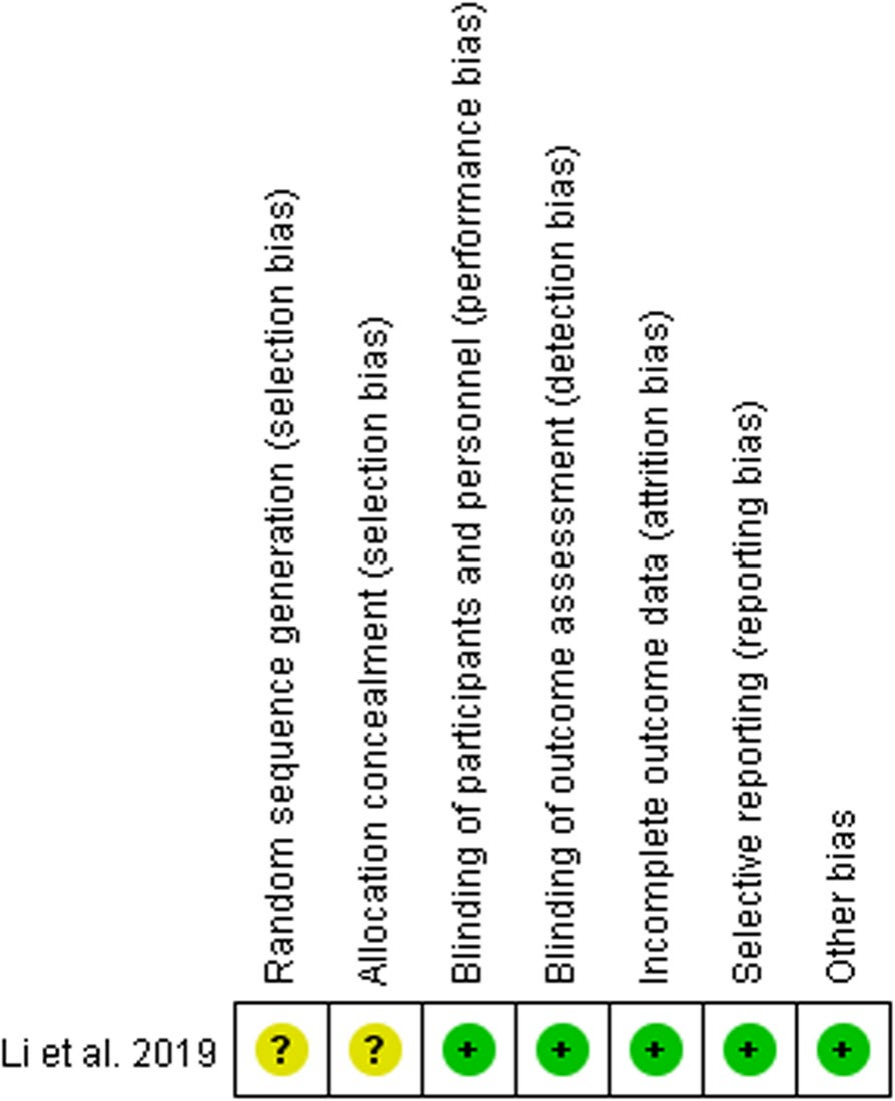
Risk of bias summary for the included RCT

The methodological quality of the RCT assessed based on Cochrane Collaboration’s tool is presented in Fig. 2. The study [36] had selection bias assessed as unclear without an adequate randomization procedure and was considered as unclear risk of bias overall. NOS was applied to investigate the risk of bias of the residual five observational studies [27, 33–35, 37]. All of the identified studies had relatively low risk of bias with NOS scores≥6. More details were presented in an additional table [see Supplementary Table 1].

### Clinical efficacy

#### Survival

**Fig. 3:**
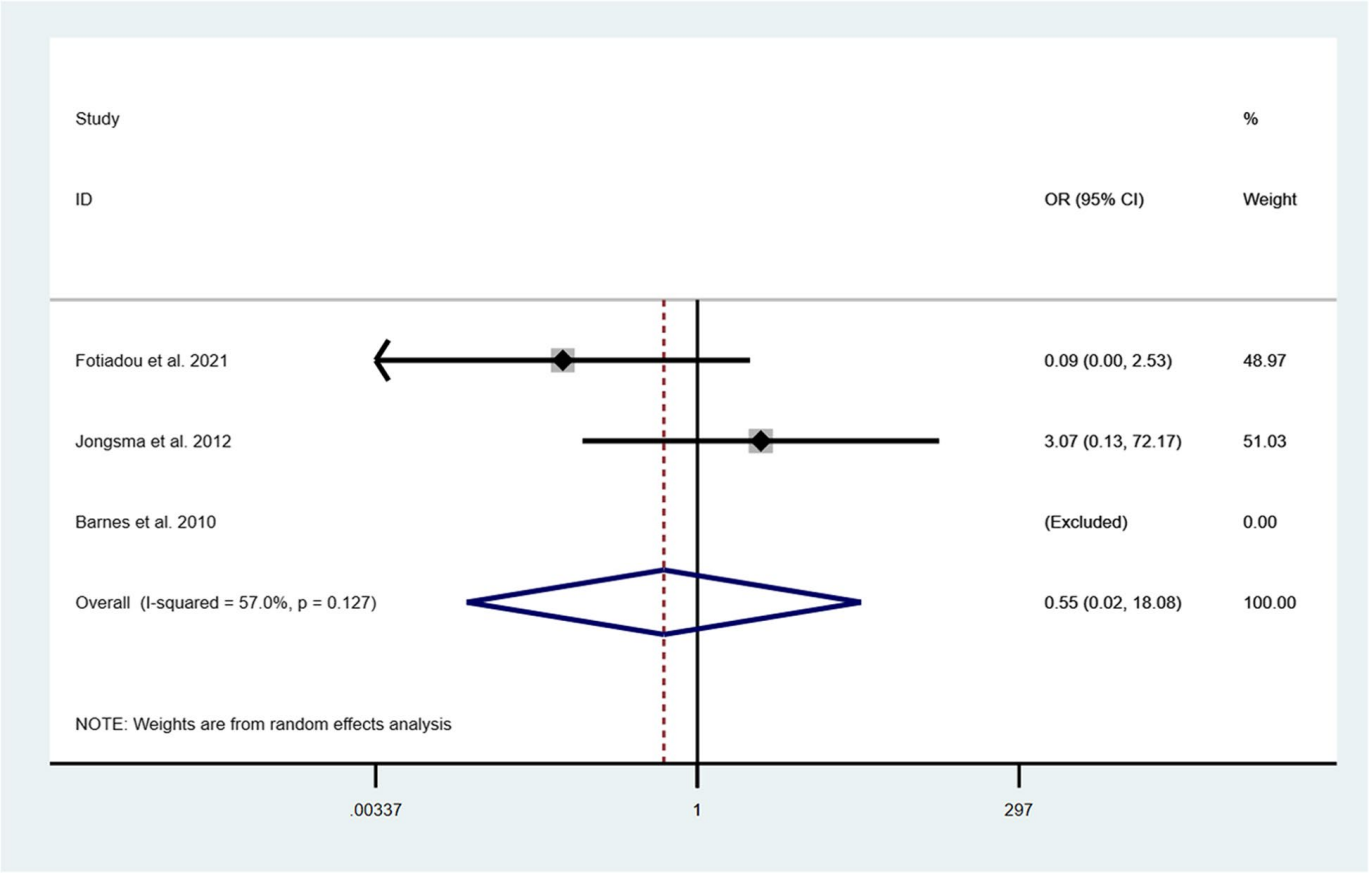
Comparison of onlays/partial crowns and crowns regarding the survival after 1 year

**Fig. 4:**
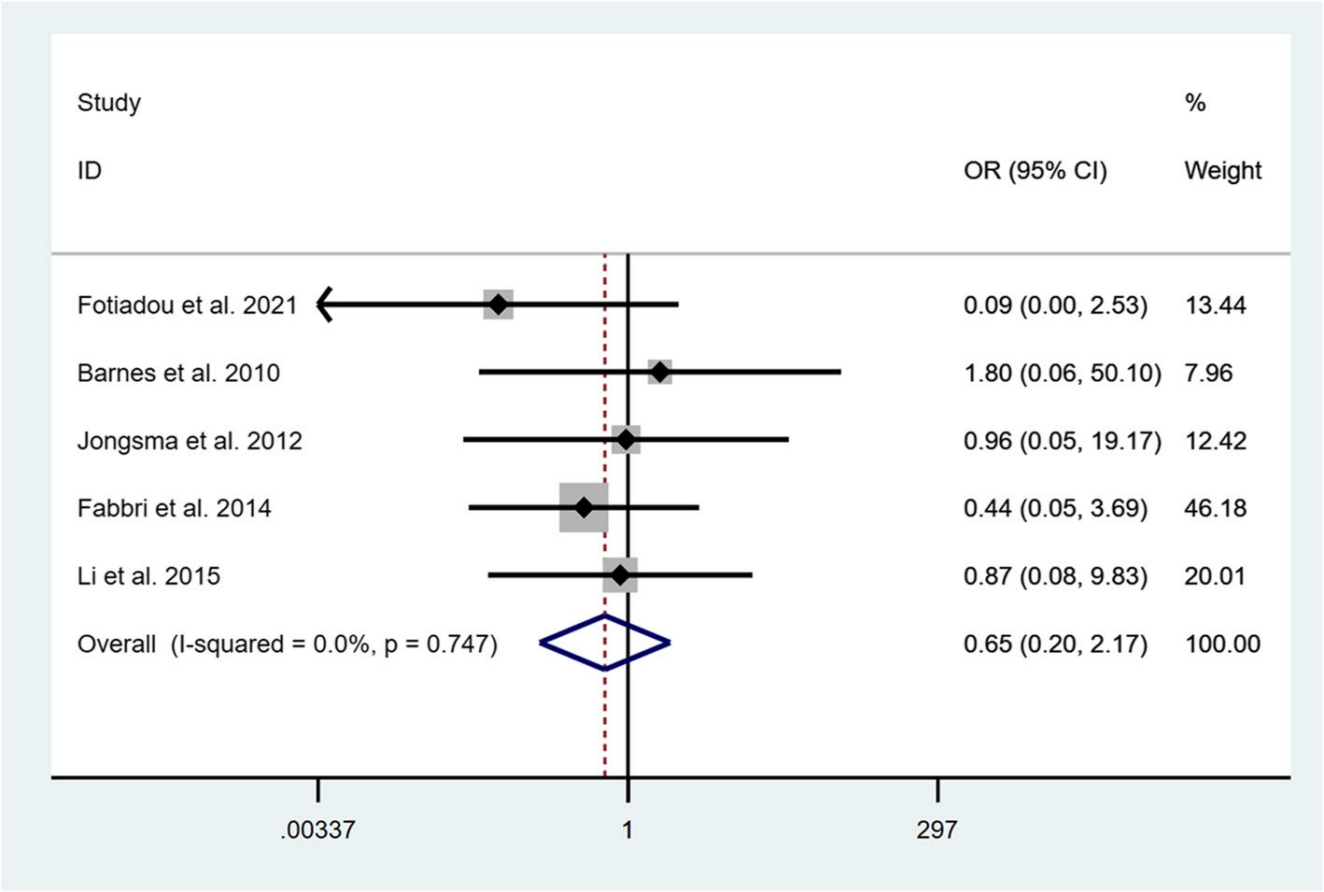
Comparison of onlays/partial crowns and crowns regarding the survival after 3 years

Five studies [33–37] reported the clinical outcomes of the onlays/partial crowns and full crowns after 1 year of follow-up. The RCT [36] showed the excellent performance of both restorations without any failures after 1 year of clinical observation. The other four studies [33–35, 37] presented the satisfactory survival rates of the onlays and crowns as well. The survival presented an OR of 0.55 (95% CI: 0.02-18.08; I^2^ = 57.0%; *P* = 0.127) in favor of onlays/partial crowns after 1 year of follow-up (Fig. 3). However, it was not statistically significant. At 3 years, OR increased to 0.65 (95% CI: 0.20-2.17; I^2^ = 0.0%; *P* = 0.747) (Fig. 4), which meant that the survival rate of onlays/ partial crowns was still higher than that of full crowns. Furthermore, Fotiadou et al. [37] investigated the longevity of both types of restorations after a mean observation of 6.6 years, pointing out that the survival rates of ceramic onlays/ partial crowns and full crowns were 96.1 and 80.0%, respectively.

#### Success

**Fig. 5:**
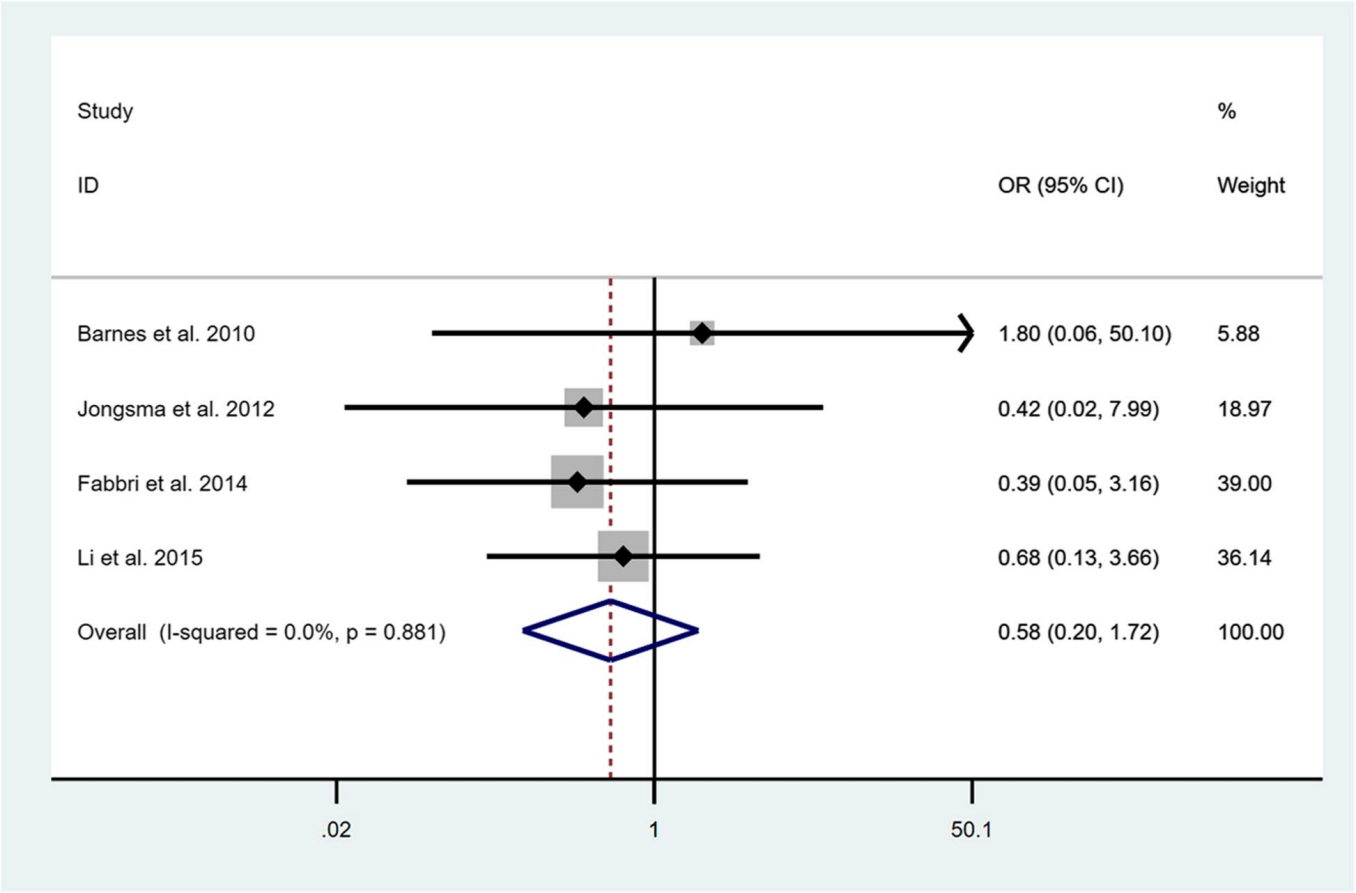
Comparison of onlays/partial crowns and crowns regarding the success after 3 years

According to the included studies, one RCT [36] and two observational studies [33, 34] reported the success rates of onlays/partial crowns and crowns after 1 year in service. Li et al. [36] reported that among 38 crowns, 5 showed defects while 3 of 36 onlays did not perform excellently. Barnes et al. [33] showed that no defects were observed in neither ceramic onlays nor crowns. Jongsma et al. [34] demonstrated that all of composite onlays functioned perfectly while 10.5% of crowns had complications after 1 year of clinical observation. Due to different study type and insufficient studies, OR was not calculated after 1 year. Four studies [27, 33–35] were kept for OR analysis for the onlays/partial crowns in comparison with the crowns at 3 years of follow-up. Based on the four studies included, onlays/partial crowns were associated with a lower OR of defects (OR = 0.58; 95% CI: 0.20-1.72; I^2^ = 0.0%; *P* = 0.881) (Fig. 5). However, it did not show a statistically significant advantage.

#### Complications

**Fig. 6:**
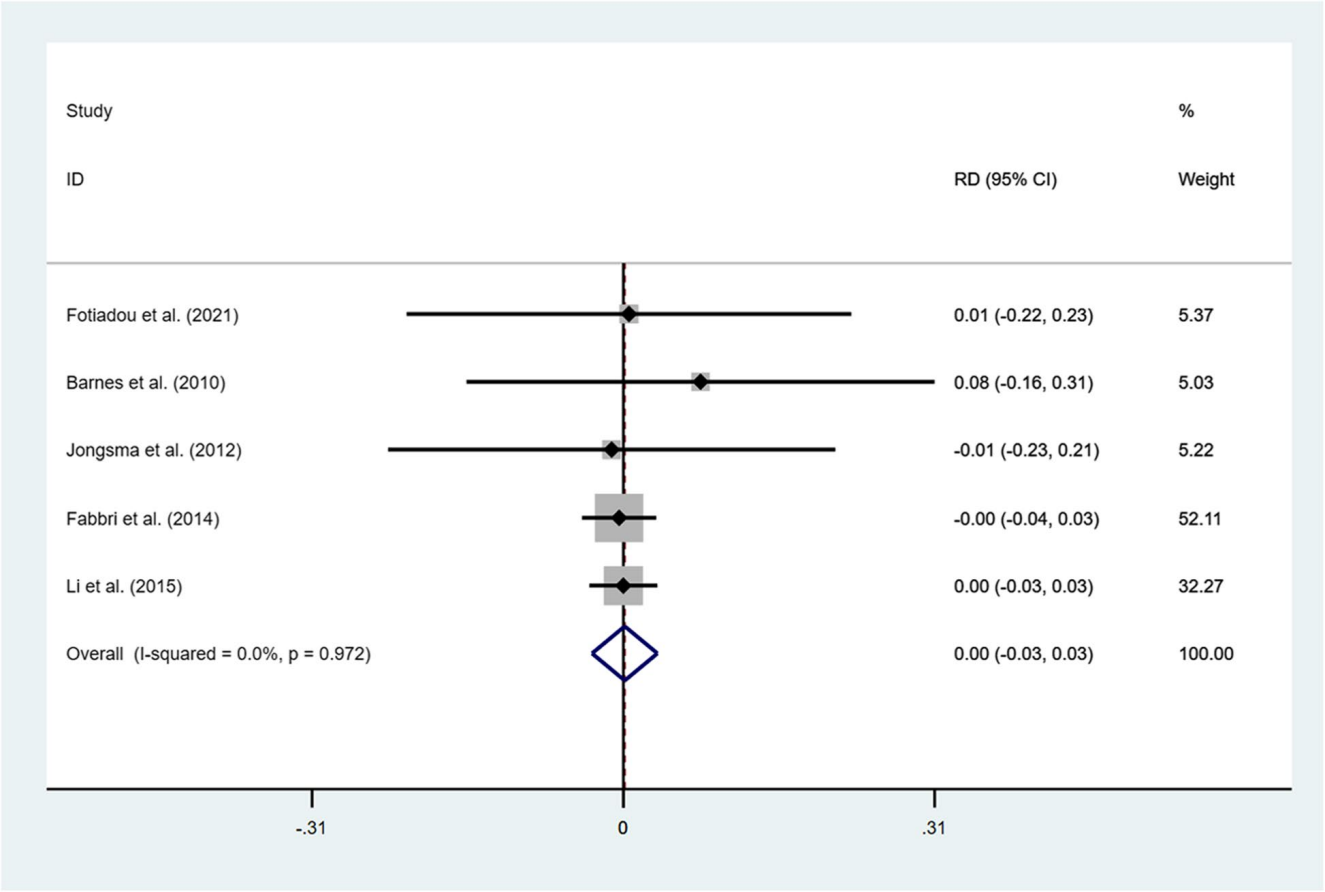
RD of crown fracture for comparison of two restorative methods after 3-year survival

**Fig. 7:**
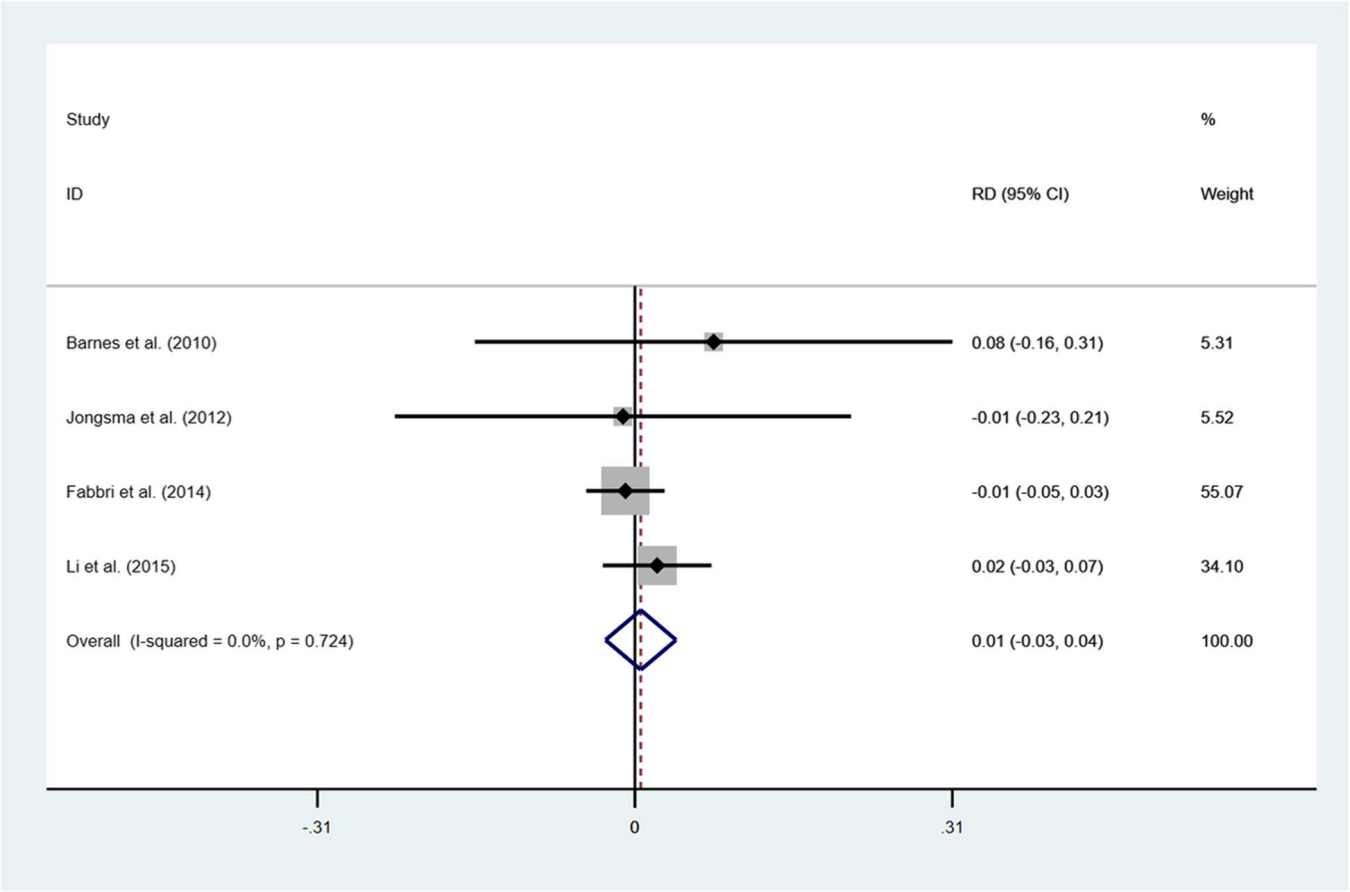
RD of crown fracture for comparison of two restorative methods after 3- year success

The failures and complications of all the selected studies are summarized in Table 2 and Table 3. According to the included studies, crown fracture, core fracture and debonding were the common mechanical complications. Pulpitis or periapical periodontitis, secondary caries, root fracture and gingivitis were the main classes of biological complications in both groups. Because of insufficient studies, we only calculated the risk difference (RD) regarding the fracture of the restorations in both groups at 3 years of clinical observation. The outcomes of RD proved that no significant difference of crown fracture was developed between the two methods regardless of the survival (RD = 0.00, 95% CI: − 0.03-0.03; I^2^ = 0.0%; *P* = 0.972) or the success (RD = 0.01, 95% CI: − 0.03-0.04; I^2^ = 0.0%; *P* = 0.724) (Fig. 6 and Fig. 7).

**Table 2:** Failures of the identified studies

No.	Author (year)	Materials	Follow-up period (y)	Failures / Total		Crown fracture	Pulpitis or periapical periodontitis	Secondary caries	Root fracture	Core fracture	Debonding	Root canal perforation
1	Li et al. (2015) [27]	IPS Emax press PFM (gold)	3	onlays: crowns:	1 / 46 2 / 86		1	1				1
2	Barnes et al. (2010) [33]	Finess all-ceramic	3	onlays: crowns:	1 / 13 0 / 7	1						
3	Jongsma et al. (2012) [34]	Neco	3	onlays: crowns:	0 / 5 7 / 86	1			1	4	1	
4	Fabbri et al. (2014) [35]	IPS Emax press	3	onlays: crowns:	1 / 62 7 / 197	1 4			1		2	
5	Li et al. (2019) [36]	IPS Emax CAD	1	onlays: crowns:	0 / 38 0 / 40							
6	Fotiadou et al. (2021) [37]	IPS Emax press	6.6	onlays: crowns:	7 / 179 1 / 5	4 1	1	2

**Table 3:** Complications of the identified studies

No.	Author (year)	Materials	Follow-up period (y)	Complications / Total		Crown fracture	Pulpitis or periapical periodontitis	Secondary caries	Root fracture	Core fracture	Debonding	Root canal perforation	Proximal contact	Gingivitis	margin adaptation
1	Li et al. (2015) [27]	IPS Emax press PFM (gold)	3	onlays: crowns:	2 / 46 5 / 86	1	1	1			3	1			
2	Barnes et al. (2010) [33]	Finess all-ceramic	3	onlays: crowns:	1 / 13 0 / 7	1									
3	Jongsma et al. (2012) [34]	Neco	3	onlays: crowns:	0 / 5 15/ 86	1	3		1	6	4				
4	Fabbri et al. (2014) [35]	IPS Emax press	3	onlays: crowns:	1 / 62 8 / 197	1 5			1		2				
5	Li et al. (2019) [36]	IPS Emax CAD	1	onlays: crowns:	3 / 38 5 / 40	1					1		1	1 2	2

#### Publication bias analysis

**Fig. 8:**
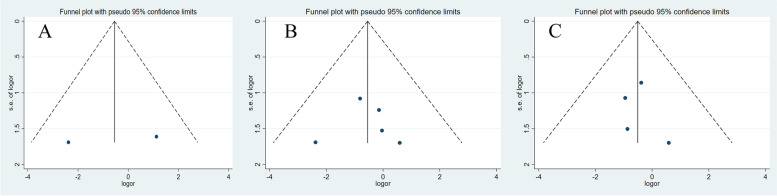
Funnel plots of the included studies. A, 1-year survival; B, 3-year survival; C, 3-year success

The publication bias of the meta-analysis was estimated using funnel plots. As shown in Fig. 8, no obvious asymmetry was revealed from the shape of the funnel plots. Furthermore, Egger’s test confirmed that no publication bias existed among the selected studies (*P* > 0.05).

## Discussion

The present review mainly evaluated the clinical efficacy of onlays/partial crowns when compared with full crowns. On the basis of clinical trials, several systematic reviews have compared the clinical efficacy of posterior indirect minimally invasive restorations with crowns [9, 38]. However, no review concerning the direct comparison of onlays/partial crowns and full crowns was found. Al-Haj Husain et al. [38] investigated the clinical outcomes of partial and full-coverage restorations fabricated from hybrid polymer and ceramic CAD/CAM materials. They evaluated the performance of the restorations in regard to the biologic, technical and esthetical aspects, and calculated the survival ratios and success rates of partial coverage restorations (inlays, onlays, overlays) and full crowns separately according to the different included studies. Finally, they concluded that the biologic, technical and esthetic success rates of partial crowns were lower than that of full crowns [38]. Vagropoulou et al. [9] compared the survival rates and complications of inlays and onlays with full crowns to identify the better type of indirect restorations in restoring single teeth. The mean 5-year survival rate of inlays was up to 90.89% while the rates of onlays and crowns reached 93.50 and 95.38%, respectively. However, no meaningful comparison between types or restoration of materials could be made due to the heterogeneity of the identified studies [9]. In this review, we assessed the odds ratios (OR) for onlays/partial crowns and classical full crowns regarding the survival and success rates and made meta-analysis to identify whether the partial coverage restorations perform as well as traditional crowns. Based on the data in the review, we hold the view that no significant difference exists between the two methods and the outcomes of OR are confirmed that. For the survival rate, onlays/partial crowns presented an OR of 0.55 (95% CI: 0.02-18.08; I^2^ = 57.0%; *P* = 0.127) and 0.65 (95% CI: 0.20-2.17; I^2^ = 0.0%; *P* = 0.747) after 1 and 3 years of follow-up, which meant that onlays/partial crowns had lower failure rate in comparison of crowns although it was not statistically significant. As to the success, OR for onlays/partial crowns and crowns was 0.58 (95% CI: 0.20-1.72; I^2^ = 0.0%; *P* = 0.881). The results indicated that onlays/partial crowns could be alternatives to full crowns in restoring the posterior teeth.

The previous studies are in agreement with our findings [9, 26, 39, 40]. Malament & Socransky [26] reported that the estimated annual risk of failure regarding onlay restorations was 0.99% while the annual failure rate of crowns was 2.40%. klink & Huettig [39] evaluated the complications and survival of Mark II (Vita zahnfabrik, Bad Säckingen, Germany) restorations after 4 years. They drew the conclusion that partial crowns performed better than full crowns in the posterior region [39]. Sulaiman et al. [40] also found that the 4-year survival rates of IPS e.max inlays/onlays and single crowns were 98.99 and 98.85%, which meant that IPS e.max inlays/onlays had the similar survival rate with crowns. In recent, Vagropoulou et al. [9] indicated that the 5-year survival rates of onlays and crowns were up to 93.50 and 95.38%, revealing that onlays was an alternative to restore the posterior teeth.

### Long-term survival rates of onlays/partial crowns and crowns

In the study, we compared the clinical outcomes of onlays/partial crowns with full crowns up to the mean follow-up period of 6.6 years. Nevertheless, the comparison of long-term survival and success rates with the follow-up period of more than 6.6 years was lacking in the review. Amounts of studies have confirmed the long-term clinical outcomes of crowns regardless of PFM or all-ceramic crowns [41–46]. Walton [42] suggested the excellent clinical performance of PFM single crowns with the estimated survival rates being 97.1 and 85.4% after 10 years and 25 years, respectively. Malament et al. [45, 46] showed that the 10-year and 16.9-year cumulative survival rates of e.max crowns were up to 99.6 and 96.75%. Moreover, Olley et al. [44] reported that the survival rate of metal-ceramic crown restorations was 96.1% and all of the ceramic crowns functioned well after 50 years. Meanwhile, ceramic onlays/partial crowns were also proved as reliable restorations after 10 years of follow-up [47–50]. Stoll et al. [47] pointed out that the survival rate of partial crowns made of IPS Empress (Ivoclar Vivadent, Schaan, Liechtenstein) in posterior region was 90.2% after a 10-year observation. Malament et al. [48] demonstrated that 98.3% of the IPS e.max onlays functioned well in posterior teeth even after 10.9 years, providing excellent clinical outcomes. Edelhoff et al. [49] investigated the clinical performance of IPS e.max occlusal onlays in patients with severe tooth wear at 11 years and found that the survival rate of onlays reached 100%, presenting exceptional results without any secondary caries, biological complication, or debonding. van Dijken and Hasselrot [50] demonstrated that the survival rate of 252 IPS Empress partial coverage restorations was 75.9% after 15 years.

### How is the performance of onlays/ partial crowns restoring the severely defected teeth?

Christensen [51] suggested that onlays were indicated when the width of the isthmus exceeded 1/2 distance from buccal cusp tip to lingual cusp tip and/or when weak cusp existed. Ferraris [1] also suggested the application of onlays in the cases that medium- to large-size cavities involving one or more cusps existed. Furthermore, several clinical trials investigated the clinical outcomes of ceramic onlays/partial crowns restoring the severely defected teeth [27, 52–55]. van Dijken et al. [52] investigated the extensive dentin/enamel-bonded ceramic partial coverage restorations and found that the survival rate was more than 90% after 5-year follow-up, indicating another promising method in restoring posterior teeth with extensive tooth substance loss. Reich et al. [53] reported that the survival rate of large all-ceramic onlays repairing at least one cusp and half of the occlusal surface was up to 94.1% at the 3-year recall appointment, demonstrating that the adhesively luted ceramic onlays could restored large coronal defects successfully. Roggendorf et al. [55] confirmed that even severely decayed teeth with at least one cusp and half of the occlusal surface missing could be successfully restored by onlay restorations as well. Li et al. [27] suggested that the survival and success rates of the severe defect (mesial-occlusal-distal defect) premolars restored by IPS e.max onlays reached 100% and 94.1% while the survival and success rates of the severely defected premolars repaired using fiber posts and full crowns were 96.2% and 88.5% after 3 years, respectively.

### Fracture resistance - how to choose materials?

According to the included studies, crown fracture, core fracture and debonding were the main technical complications. The principal complication of failures was crown fracture in both types of restorations. All of the identified studies reported the fracture of restorations. Among the 27 failures, a total of 12 restoration fracture occurred with 6 failures in the onlay group and 6 in the crown group, which meant ceramic onlays and crowns may have the similar fracture rate. RD regarding the fracture of restorations proved it. In fact, we found out two in vitro studies comparing the difference of fracture resistance between onlays/partial crowns and full crowns [56, 57]. Yu et al. [56] investigated the fracture resistance and fracture modes of IPS e.max onlays and crowns, finding that ceramic onlays and crowns had the similar fracture resistance. Nevertheless, mode II (less than half of the crown is lost) was the main fracture type of onlays restoring the endodontically treated teeth while severe fractures (Type V, a severe fracture of the crown and/or tooth) occurred mainly in the endodontically treated teeth restored by crowns [56]. Frankenberger et al. [57] reported that no significant difference was found between the partial crowns and full crowns in the e.max group regarding post-fatigue fracture resistance. These laboratory outcomes were in agreement with our study. Additionally, the material type also affected the fracture resistance of the onlays and crowns. Although IPS e.max onlays/partial crowns had the similar performance on fracture resistance when compared to the classical crowns, zirconia partial crowns exhibited significantly lower fracture resistance than full crowns [57].

On the other hand, it is still controversial whether IPS e.max onlays/partial crowns perform better than zirconia onlays/partial crowns in the laboratory studies. Frankenberger et al. [57] confirmed that zirconia partial crowns had the lower fracture resistance than e.max partial crowns without significant difference. Contrarily, Saridag et al. [58] showed that zirconia onlays had significantly higher fracture resistance than e.max onlays. Nevertheless, the fracture of zirconia onlays mostly extended to the tooth while the fracture in e.max samples were restricted to the restoration itself in general [58]. The result was also supported by Wafaie et al. [59] However, Mynampati et al. [60] reported that zirconia onlays had stronger fracture resistance than e.max onlays and the fracture modes of zirconia onlays were also much safer than e.max onlays. Due to the difficulty of adhesive and cementation [3], no clinical study of zirconia onlays/partial crowns is published. Hence, no comparison of clinical outcomes between zirconia and lithium disilicate onlays/partial crowns could be made.

On the basis of the fracture resistance and mode, combining the in vitro studies and clinical trials we systematically searched above, we draw the conclusion that IPS e.max seems to be more suitable for onlays/partial crowns while zirconia performs better for crowns. The conclusion was consistent with the study by Belli et al. [61] They found that IPS e.max onlays performed significantly better than e.max crowns. On the other hand, monolithic ZrO2 crowns performed better than e.max crowns although the ZrO2 crowns were followed only for a short evaluation period [61]. Due to the lack of clinical studies of zirconia onlays/partial crowns, no direct comparison could be made between the zirconia onlays/partial crowns and other restorations.

Among the selected studies, Jongsma et al. [34] suggested that no significant difference of crown fracture was seen between the composite partial crowns and full crowns. We did not find out any laboratory studies that directly compared the fracture resistance between composite onlays/partial crowns and full crowns. Several clinical trials reported the excellent performance of composite onlays [62–65]. But, Kois et al. [66] evaluated the performance of ceramic and resin-based composite partial coverage restorations in posterior region and pointed out that teeth with composite onlays were more inclined to the extensive fractures involving tooth and root structure. The result was in accordance with the study by Gomes de Carvalho et al. [67] Gomes de Carvalho et al. [67] found that composite onlays led to a higher stress concentration as well as higher peaks in the dental structure and were more prone to result in tooth fracture. Li et al. [68] also pointed out that when compared to IPS e.max onlays, Lava ultimate onlays resulted in higher risks of irreparable failures due to high stress concentrations in the shoulder of teeth. So were composite crowns. Campos et al. [69] reported that all fractures of polymer full crowns (Targis) extended to the root in a catastrophic manner as well, resulting in the limited use of polymer crowns in clinics.

### Fracture resistance - how to prepare the tooth restored with onlays/partial crowns?

In the premolar region, the in vitro studies were more in favor of onlays with total cuspid coverage regardless of materials [70–74]. IPS Empress II, e.max and Vitadur Alpha ceramic (Vita Zahnfabrik, Bad Sackingen, Germany) onlays with total cuspid coverage had higher fracture resistance than onlays with palatal cuspid coverage though there was no significant difference between the two different preparation designs [70–72, 74]. In addition, Harsha et al. [73] reported that zirconia onlays with total cuspal coverage had significantly stronger resistance to fracture than zirconia restorations with only palatal cuspal coverage. As to fracture mode, the results were not always consistent. Stappert et al. [70] and Harsha et al. [73] held the view that onlays with complete cuspal coverage showed safer and more restricted fracture modes while Cubas et al. [72] demonstrated the contrary result. Cubas et al. [72] reported that most of the onlays with palate cuspal coverage presented a fracture restriction to the restoration while onlays with both cuspal coverage were more prone to fracture both in the restoration and tooth. In the molar region, different in vitro studies compared the preparations of different cuspal coverage. Stappert et al. [75] investigated the fracture resistance of onlays/partial coverage restorations (PCR) with four different preparations in maxillary molars, including mesiopalatal cuspal coverage, both palatal cuspal coverage and both palatal and distobuccal cuspal coverage as well as all cuspal coverage. They found that no significant difference was observed regarding the fracture strength values among the four groups, indicating that the different preparation designs of the PCRs had little statistically significant influence on the fracture resistance of the restorations [75]. Later, Stappert et al. [76] reported that onlays with buccal cusp coverage in mandibular molars had the similar fracture resistance with restorations with total cusp coverage as well. Soares et al. [77] reported that onlays involving only mesio-buccal cusp coverage showed the lowest mean fracture strength value (1612.2 N) in the mandibular molars while onlays with buccal cusp coverage had the highest mean fracture strength value (2158.4 N). Significant difference was identified between the different preparations with fractures generally restricted to the restorations [77]. Further in vitro studies and clinical trials are needed to identify the best preparation of onlays/partial crowns in the molar region.

### Biological complications

Pulpitis or periapical periodontitis, secondary caries, root fracture and gingivitis were the main classes of biological complications in both groups. In general, for a mount of the sound tooth structure removal, endodontic complications are more prone to occur in the full crowns [78]. Benefited by less tooth removal, onlays/partial crowns possess the advantages of protecting the pulp’s health, facilitating visual margin control and easier performance of oral hygiene for patients [75]. However, due to a longer finishing line than the corresponding full crowns, teeth with onlays were susceptible to recurrent decay [9]. Nevertheless, the association between the two kinds of complications (secondary caries and endodontic complications) and the types of restorations (onlays and crowns) was not consistent by different studies. Abduo & Sambrook [10] reported that the principle biological complication of ceramic onlay failures was caries. Bustamante-Hernandez et al. [79] also concluded that caries was the most frequent biological complication of ceramic, hybrid and composite onlays. Fan et al. [80] demonstrated that the most frequent reason of posterior composite indirect restorations leading to failures was secondary caries with the polled proportion of 47% among the failures at 5-year follow-up. However, for ceramic restorations, endodontic complications were the main biological complication with the cumulative proportion of 20% among the failures [80]. As to full crowns, Gehrt et al. [81] found that the most frequent biological complication was endodontic infection. The outcome was also proved by Pjetursson et al. [82] They claimed that the predominant biological complication was loss of pulp vitality (0.43 per year). In addition, Larsson & Wennerberg [16] also held the view that endodontic treatment was the most common biological reason of crowns. On the contrary, Vagropoulou et al. [9] revealed that the main biological complication of crowns was caries, followed by tooth fracture and endodontic reasons. In our review, due to low failure rates and excellent performance of both groups, the definite relationship between the two complications and types of restorations could not be confirmed.

### Limitation of this systematic review and meta-analysis

Most of the data in the included studies reveal homogeneity. Among six studies adopted in this review, only one study is RCT while five are prospective or retrospective observational studies with their evidence levels being lower than that of RCTs. The evaluation criteria in the selected studies for restoration assessment was not the same, though it may not affect the data power. In addition, the studies adopted showed the short-term clinical outcomes of onlays/partial crowns and full crowns. Hence, further RCTs are called for to investigate the long-term clinical outcomes of onlays/partial crowns compared to full crowns.

## Conclusion

Overall, within the limitations of the review, onlays/partial crowns appeared to function as well as full crowns regardless of the survival or success after the short-term follow-up period. When analyzing the crown fracture, no difference was found between the two restorative methods. However, high-quality RCTs with long-term follow-up are recommended to consolidate the efficacy of onlays/partial crowns in the posterior region.

## Supplementary Information

**Additional file 1: Supplementary Table 1.** NOS of the observational studies.

## Data Availability

The datasets used and analyzed for the current study were available from the corresponding author upon reasonable request.

## Abbreviations

RCTs: Randomized-controlled trials
OR: Odds ratio
CI: Confidence interval
I^2^: Inconsistency index
RD: Risk difference
PCCs: Partial ceramic crowns
PRISMA: Preferred Reporting Items for Systematic Reviews and Meta-Analyses
NOS: Newcastle-Ottawa Scale
PFM: Porcelain-fused-metal
PCR: Partial coverage restoration
